# ELAN: A Software Package for Analysis and Visualization of MEG, EEG, and LFP Signals

**DOI:** 10.1155/2011/158970

**Published:** 2011-04-20

**Authors:** Pierre-Emmanuel Aguera, Karim Jerbi, Anne Caclin, Olivier Bertrand

**Affiliations:** ^1^INSERM U1028, CNRS UMR5292, Lyon Neuroscience Research Center, Brain Dynamics and Cognition Team, Centre Hospitalier Le Vinatier, Bâtiment 452, 95 Boulevard Pinel, 69500 Bron, France; ^2^University Lyon 1, 69000 Lyon, France

## Abstract

The recent surge in computational power has led to extensive methodological developments and advanced signal processing techniques that play a pivotal role in neuroscience. In particular, the field of brain signal analysis has witnessed a strong trend towards multidimensional analysis of large data sets, for example, single-trial time-frequency analysis of high spatiotemporal resolution recordings. Here, we describe the freely available ELAN software package which provides a wide range of signal analysis tools for electrophysiological data including scalp electroencephalography (EEG), magnetoencephalography (MEG), intracranial EEG, and local field potentials (LFPs). The ELAN toolbox is based on 25 years of methodological developments at the Brain Dynamics and Cognition Laboratory in Lyon and was used in many papers including the very first studies of time-frequency analysis of EEG data exploring evoked and induced oscillatory activities in humans. This paper provides an overview of the concepts and functionalities of ELAN, highlights its specificities, and describes its complementarity and interoperability with other toolboxes.

## 1. Introduction

The unprecedented increase in computational power over the last 20 years has led to extensive developments of advanced data processing and visualization methods. The steady stream of novel methods that continue to flourish in the field of electrophysiological data processing has substantial implications in numerous fields including basic neurophysiology, cognitive brain research, and clinical neuroscience. Indeed, in recent years, noninvasive recordings techniques such as electroencephalography (EEG) or magnetoencephalography (MEG), and invasive methods, such as intracranial EEG (iEEG) or microelectrode recordings (measuring local field potentials, LFPs) increasingly rely on sophisticated analysis of high-resolution data sets, across multiple spatial, temporal, and spectral dimensions. Yet, beyond the attractive perspective of applying emerging methods to one's data, many researchers and clinicians face the question of method development and software selection. In this context, sharing existing methods via freely available software packages and toolboxes is poised to play an important role, especially when combined with a user-friendly environment and computationally efficient code.

The ELAN software package provides a wide range of preprocessing and signal analysis tools for many types of electrophysiological data. Data types typically analyzed with ELAN includes macroscopic surface-level recordings such as scalp electroencephalography (EEG) or magnetoencephalography (MEG), invasive recordings such as intracranial EEG (iEEG) in humans (electrocorticographic, ECoG, and stereotactic EEG) as well as local field potentials (LFPs) in animals. In addition to many basic signal processing tools for electrophysiological data, the main features of ELAN include topographical mapping, time-frequency analysis, along with adapted statistical routines, and interactive user-friendly visualization tools to navigate in the data sets, all implemented in C for fast computations.

The first version of the ELAN toolbox was released in 2001, integrating 15 years of methodological developments at the INSERM Brain Dynamics and Cognition laboratory in Lyon and has served as the standard in-house software for the analysis of all types of electrophysiological data. So far ELAN has been used by direct collaborators and was licensed to approximately 20 national and international research labs. To date, the methods incorporated in ELAN have been used and reported in over 100 publications across various fields in neuroscience and electrophysiology. With the publication of this paper, the ELAN software package will become available for free download. The developers of ELAN aim to create a community of users and a knowledge base for exchange of expertise and continued development and improvement of the ELAN software package in the future.

The goal of this paper is to provide an overview of the concepts and functionalities that build the structural backbone and strengths of ELAN. We also highlight the specificities of this software and describe its complementarity and interoperability with other existing toolboxes. Up-to-date information about downloads, user guide, tutorials and additional resources are available online at the ELAN website (http://elan.lyon.inserm.fr/). 

## 2. ELAN Software: Concepts and Functionalities

In this section we describe the main features of the ELAN package including system requirements, overall data workflow, basic ELAN analysis tools and visualization modules, as well as compatibility and interoperability issues.

### 2.1. System Requirements

ELAN is available for 32-bit and 64-bit Linux operating systems (Debian, Fedora, and OpenSuse distributions are currently supported and therefore most of their derivative implementations as well, including Ubuntu and CentOS). Mac and Windows users can also run ELAN on their system by first installing a virtual machine that runs a Linux guest in their preferred host (examples of such virtualization software include VirtualBox and VMWare). The minimal RAM memory requirement is 128 MB, but 4 GB is highly recommended. The ELAN toolbox can import data from many commercially available electrophysiological data acquisition devices, including research and clinical systems. These include: Alpha Omega AlphaMap (Alpha Omega, Nazareth, Israel), Wavemetrics IGOR Pro (WaveMetrics Inc., Lake Oswego, OR, USA), Biosemi (BioSemi, Amsterdam, The Netherlands), Brain Products Recorder (BrainProducts GmbH, Munich, Germany), CTF Inc. (VSM Med Tech Ltd., Coquitlam, BC, Canada), Elekta/Neuromag (Helsinki, Finland), EGI Geodesic (Electrical Geodesics Inc., Eugene, OR, USA), InstEP System (InstEP systems, Ottawa, Canada), Micromed (Micromed, Treviso, Italy), Neuroscan (Neuroscan Inc., VA, USA). In addition, beyond standard data formats from hardware devices, ELAN can also import data by conversion from many standard file format such as ASCII (text), MATLAB (mat file), EDF (European Data Format), GDF (General Data Format for Biosignals). New data conversion and import tools are constantly added to the ELAN package.

### 2.2. ELAN's Workflow

ELAN consists of compiled C programs that make up a bundle of data analysis and visualization functions for various electrophysiological data including EEG, MEG, iEEG, LFP, reconstructed source time series, or any type of continuous signals. All ELAN functions can be run in command-line or batch-mode. Note that some routines (e.g., interfacing with EEGlab ICA routines) are implemented and provided as MATLAB functions (.m files). In the following, we describe the main data formats and visualization tools available in ELAN.

#### 2.2.1. Data and Event File Types

**Figure 1:**
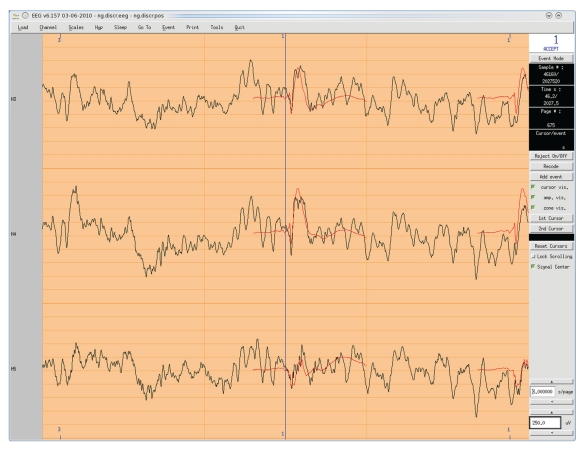
*EEG* vizualisation tool for continuous data (.eeg file), with an example of intracranial auditory EEG signals. *X*-axis: time, *Y*-axis: signal amplitude, one row per channel. Events are displayed above and below the continuous EEG signals, and the averaged event-related response is superimposed in red. Various plotting tools and online analysis functions are available through the menus (top and right of the window).

**Figure 2:**
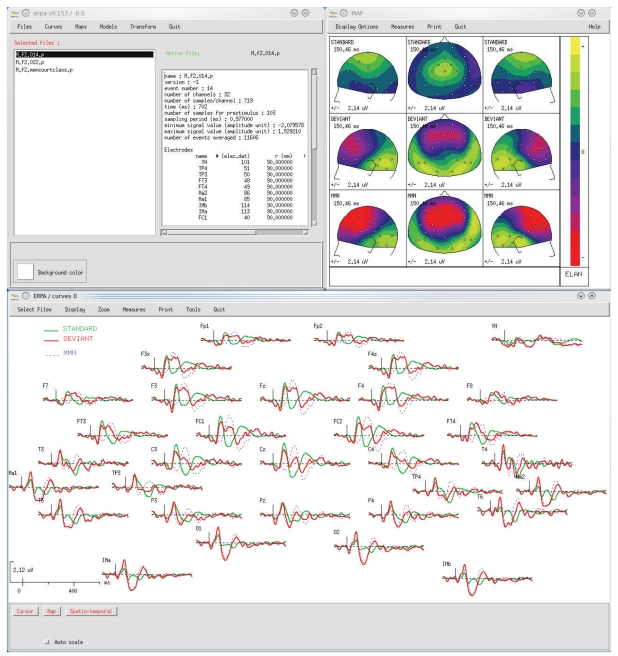
*ERPA* visualization tool for averaged signals (.p file), with an example of scalp auditory ERPs (MMN protocol). Top left window: main ERPA window to select files and launch visualization tools. Bottom: time courses of event-related responses (positivity is up). Top right: mapping of event-related responses, here with several views of three different ERPs at the same latency. In the windows where data are visualized, plotting functions and tools for on-the-fly signal processing or measurements are available through the menus at the top.

**Figure 3:**
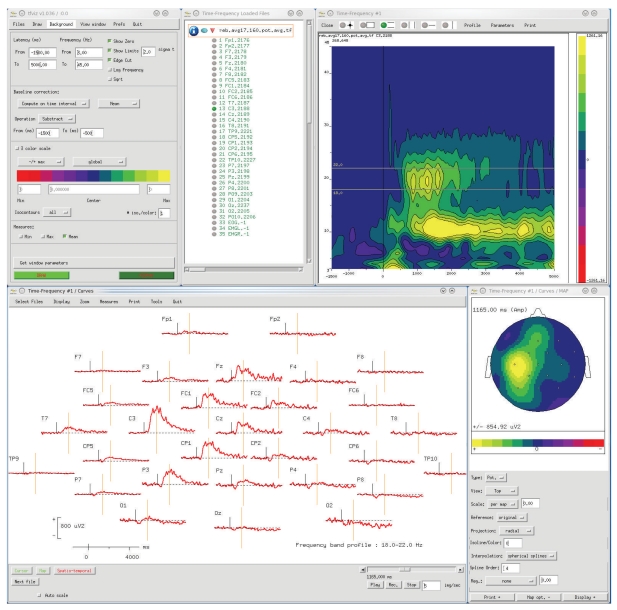
*TFVIZ* visualization tool for time-frequency data (.tf file), with an example of scalp EEG in a motor finger tapping protocol. Top left: main *TFVIZ* window, to select files, set vizualisation parameters, apply baseline correction, and so forth. Top center: window to select the channels to visualize. Top right: time-frequency plot, here for one channel in one TF file (*X*-axis: time, *Y*-axis: frequency, amplitude is color-coded). Bottom left: visualization of the time-course of signal amplitude in one frequency band defined by two cursors on the TF plot (this display offers the same functionalities as in *ERPA*, see Figure 2). Bottom right: topography for one frequency band at the latency defined by the cursor on the curves. This figure illustrates the possibility to navigate with *TFVIZ* in this multidimensional data set (in the frequency, time, and spatial dimensions).

ELAN uses three main data formats: continuous data (.eeg, see Figure 1), event-related data (.p, see Figure 2), and time-frequency data (.tf, see Figure 3). First, the continuous data format (.eeg) stores any continuous data with event codes corresponding to stimulations and behavioral events such as experiment triggers and subject's responses. The continuous data can be EEG, MEG, iEEG, LFP (for examples of animal datasets analysis, see [1, 2]), reconstructed source time series, ICA components, EEG Laplacians (see Section 2.3.2 below), and so forth. Second, ELAN event-related data format (.p) typically stores data that has been averaged with respect to a specific event. However, this same format can also be used to process temporal or frequency profiles of various types. Finally, the time-frequency (.tf) data format files store the output of all time-frequency analysis functions available in ELAN. 

In addition to the three main data types, ELAN uses two other types of files: event files and parameter files. Event files are in standard text format (.pos) and store all information related to the events (e.g., triggers, responses, etc.) that occur in a given continuous data set (.eeg). The event file can easily be modified in order to reject, group, recode, or create new events. 

Furthermore, a typical call to an ELAN function requires the specification of a number of parameters. These computing parameters are stored and passed to the function via a parameter text file (.par). A parameter file may, for example, contain flags indicating which channels to use, time window parameters (with respect to specified events), parameters for the time-frequency analysis or for the statistical analysis, and so forth.

An important feature of the ELAN toolbox is that the result of any analysis performed with an ELAN function is always stored in one of the three main data formats (.eeg,.p, or.tf), so that it can still be processed and visualized using ELAN tools. For example, the time profile of a particular frequency band in a time-frequency file (.tf) can be extracted and then visualized and/or analyzed as an event-related data file (.p). Similarly, reconstructed time-courses of neural generators obtained after source modeling with another software and converted to an ELAN file format can be analyzed in the time-frequency domain just as original scalp recordings.

#### 2.2.2. Data Visualization

ELAN contains three distinct data visualization graphical user interfaces (GUIs). In principle data from each one of the three ELAN file formats (see above) is visualized with its dedicated tool. The *EEG* tool allows for the visualization of continuous data (Figure 1), *ERPA* is the tool used for event-related data files (Figure 2), while *TFVIZ* is the tool used to display the results of the time-frequency analysis (Figure 3). These three viewers share numerous common functions, such as curve plotting, cursor-based measures, topographical mapping, and so forth.

### 2.3. Overview of ELAN Tools

#### 2.3.1. Data Preprocessing

A number of ELAN tools allows for preprocessing of continuous (.eeg) data, typically performed just after importation of raw data and prior to the main analysis, to handle artifacts, filter the data, organize event codes, or even score sleep stages. 

**Figure 4:**
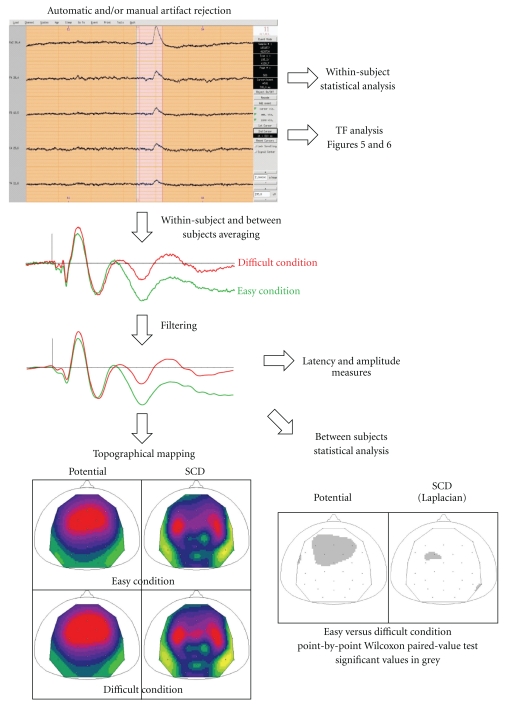
Overview of ELAN analysis workflow, with an example of scalp auditory EEG data. Artifact rejection is done automatically or manually and visualized with *EEG* (top). Signal averaging is then performed within and across subjects (middle), with a possibility to filter the data. Topography (including SCDs) can then be assessed with *ERPA* (bottom left, example of auditory N1). Note that additional frontal current sinks are identified more easily with SCDs than with potential maps. Bottom right: statistical map obtained by comparing the amplitude of the responses in two conditions across subjects at the latency of the auditory N1 (adapted from [5]).

*Artifact Rejection*. Artifacts can either be manually detected and rejected through the *EEG* visualization tool, or automatically detected and rejected, using either a fixed threshold that should not be exceeded during a trial (e.g., ±75 *μ*V), or a range of variation that should not be exceeded within a time window (e.g., 2000 fT of variation within 500 ms). Thresholds can be adjusted separately for each channel. Whenever the threshold is exceeded in at least one channel, the full trial is rejected. Information regarding whether a given trial has been kept or rejected is stored in the event file (.pos) associated with the continuous data file (.eeg) and can be visualized with *EEG* (Figure 4).

*Artifact Correction*. Ocular artifacts can be corrected for instance using ICA. This is currently possible via a provided MATLAB function that applies ICA functions to the ELAN data file using the freely available EEGLab toolbox [3]. In addition, data from bad or missing channels can be reconstructed by interpolation using spline functions.

*Data Filtering*. Continuous and concatenated epoched data (.eeg), as well as event-related data (.p), can be filtered using dedicated ELAN functions that apply Butterworth low-pass, high-pass, band-pass, or stop-band filters. In addition, any other type of filter can be applied if the user can supply its coefficients (computed with external tools such as MATLAB, Octave, Scilab, or SciPy).

*Sleep Stage Scoring*. For polysomnographic data (containing EEG, EMG, and EOG signals), ELAN allows for a convenient manual scoring of sleep stages, implemented within the *EEG* visualization tool resulting in a hypnogram file (.hyp, text file). The hypnogram can then be combined with an event file (.pos) using a MATLAB function, in order to analyze events depending on the sleep stage (e.g.,[4]).

*Event File (.pos) Preprocessing*. Prior to data averaging, a number of simple operations can be performed on event files (.pos) using ELAN functions, for example to change or regroup event codes, compute reaction times, and so forth. Furthermore, event files are simple text file that can easily be edited and processed, so that more sophisticated event preprocessing can be performed outside of ELAN, for example, using MATLAB or simply a spreadsheet.

#### 2.3.2. Time-Domain Analysis and Topographical Mapping

*Event-Related Averaging and Data Analysis.* Continuous (possibly preprocessed) data (.eeg) are averaged according to event positions stored in an event file (.pos), resulting in an event-related data file (.p) for each event code, which can be visualized using *ERPA*. Baseline correction can be performed by subtracting the average signal in a time-window either relative to the event of interest or relative to another event (e.g., averaging can be done relative to the subject's response, using a baseline taken prior to stimulus onset). Numerous operations can be performed on event-related data files (.p) using ELAN functions: filtering, (see Section 2.3.1), subtraction of two files, average of several files to obtain, for example, the grand average across subjects, and so forth. When visualizing the event-related time-courses with *ERPA*, a variety of amplitude and latency measurements can be performed and saved as text files for further analysis with a statistics software. 

*Topographical Mapping and Scalp Current Density Analysis of Event-Related Signals*. The topography of event-related EEG or MEG data (ERP, ERF) or other topographical data (ICA components, etc.) is computed through spherical spline interpolation [7] for each time point (or each component) and visualized with *ERPA* using orthogonal or radial projections. ERP mapping is done on the reference sphere of the 10–20 system. To further analyze the topography of ERP components, Scalp Current Densities (SCDs), corresponding to the surface Laplacian of the potential distribution, can be computed [8–10] and visualized with *ERPA*. SCD maps have several advantages over potential maps: they allow dissociating more easily multiple generators (see Figure 4) and are reference-free. They favor superficial generators relative to deep ones. In ELAN, SCDs can be computed on event-related data (.p) or continuous data (.eeg). In the latter case, they can be used as a preprocessing step before time-frequency analysis to dissociate the generators of the oscillatory response (e.g., [11]). It is possible to map event-related signals derived from scalp-EEG, from a whole-head magnetometer, or even from grids of electrodes placed on the cortical surface in human or animals. Finally, ELAN also incorporates specific tools to assess the spatiotemporal topography of event-related data for intracranial EEG (iEEG) acquired with depth electrodes having several contiguous contacts (e.g., see spatiotemporal mapping in [12]).

#### 2.3.3. Time-Frequency Analysis

**Figure 5:**
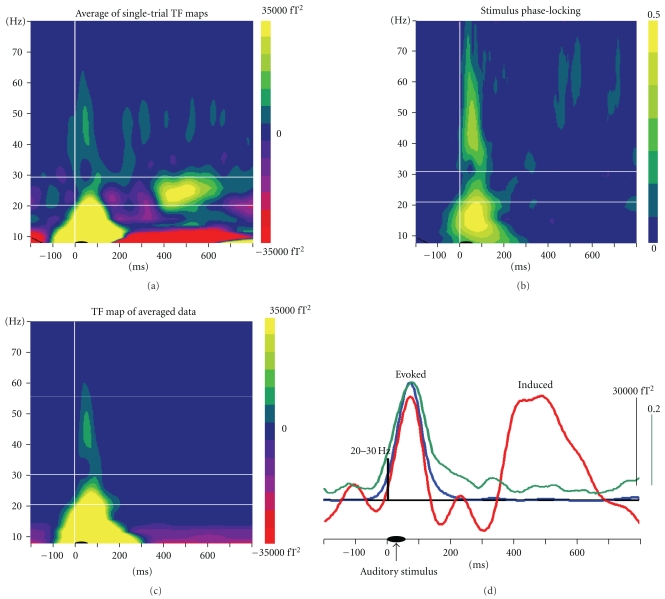
Time-Frequency analysis: dissociation of evoked and induced oscillatory activities, in an example of MEG responses to auditory stimuli. Top left: TF map obtained after averaging of wavelet transforms of single trials, which contains both evoked and induced responses. Top right: stimulus phase locking factor. For each single trial the phase of the response relative to an event (here the auditory stimulus) is computed, and the stability of the phase across trials is represented. Phase locking is close to 0 for activities not phase-locked to the stimulus also called induced activity, and larger stimulus phase locking values (up to 1) characterize stimulus phase-locked activities (evoked responses). Bottom left: TF map obtained after wavelet transform of the averaged evoked response, reflecting mostly evoked activities. Bottom right: time courses in the 20–30 Hz frequency band of the averaged oscillatory power (red), of evoked oscillatory power (blue), and of the stimulus phase-locking factor (green). The first peak of oscillatory responses at ~100 ms could be identified as evoked, whereas the second one around 500 ms is induced.

To study oscillatory brain activities, ELAN computes time-frequency (TF) representations of the signals using wavelet analysis. Typically Morlet wavelets are recommended as they provide an ideal compromise between time and frequency resolution [13]. In addition, ELAN also allows for TF analysis using Gabor transform of the signals. Generally, time-frequency power is computed for each single trial independently (using a  .eeg and a  .pos files) and then averaged across trials yielding TF maps of both stimulus-evoked and-induced activities (stored in a  .tf file). To dissociate these two components of the response, ELAN provides a number of useful functions (Figure 5). For instance, ELAN can compute a measure known as stimulus phase-locking factor (or intertrial coherence) in the TF domain (see, e.g., [14]). Besides, ELAN allows for the subtraction of the evoked response (.p files) on each single trial prior to TF transform. Furthermore, TF representations can be computed directly on the evoked response.

#### 2.3.4. Interchannel Synchrony Analysis

**Figure 6:**
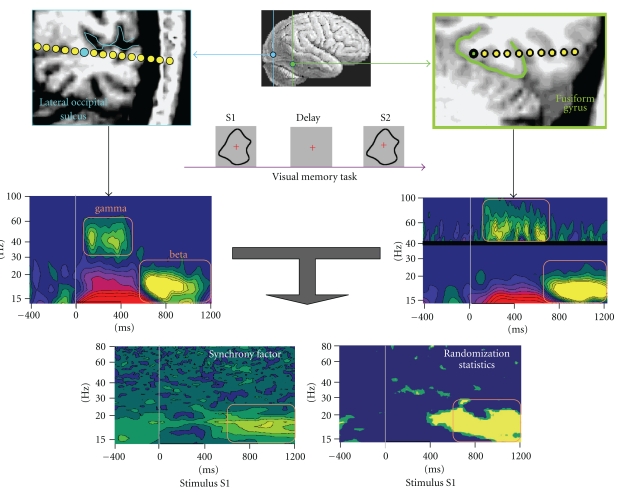
TF synchrony analysis, with an example of intracranial EEG activities after visual stimulation. Middle panels represent the TF power for two recording sites in the lateral occipital sulcus (left) and fusiform gyrus (right). Phase synchrony between the two sites was computed for each trial and averaged (bottom left). Significance of the synchrony values was assessed using randomization statistics (bottom right). Significant coupling was observed in the beta band (adapted from [6]).

ELAN can also be used to compute coupling between pairs of channels using phase synchrony measures in the time-frequency domain [15] (see, e.g., [6] and Figure 6). One advantage of phase synchrony is that it is independent of signal amplitude. However, it is sensitive to signal-to-noise ratio (SNR) of the data. Further coupling measures such as coherence and cross-frequency analysis will be added to the toolbox in future developments. Users should keep in mind the general concerns and limitations that apply to all measures of coupling, including spurious connectivity that can result from field spread at the sensor level [16].

### 2.4. Individual and Group-Level Statistics

As mentioned above, it is possible to extract the amplitudes and latencies of event-related responses (see Section 2.3.2) for statistical analysis with custom-designed routines or other commercial software. In addition, ELAN itself offers numerous possibilities to perform point-by-point statistical analysis (i.e., one statistic is computed for each sample of each channel in the time domain, and for each sample and each frequency of each channel in the time-frequency domain), both within and between subjects. The currently available statistics are mostly nonparametric and randomization tests, which have larger domains of validity than their parametric counterparts. Specific tools using randomization methods have been developed in ELAN to test multisensory interactions using an additive model [12, 17, 18].

To handle the multiple comparison problem several strategies are possible, depending on the particular test performed: Bonferroni correction, False Detection Rate (FDR) correction [19] implemented in ELAN, or applying for each channel a threshold on the minimum consecutive numbers of samples where a *P*-value lower than  .05 (or any other preset alpha level) is observed to consider an effect to be significant (for approximate values see [20]; such maximum statistics are computed in ELAN when using randomization tests). Outputs (e.g., *P*-values) of the statistical analysis programs in ELAN are always stored in an ELAN file format, and can thus be visualized using *ERPA*, or *TFVIZ*, in the temporal, spatial, and time-frequency domains.

#### 2.4.1. Across-Trial Statistics for Within-Subject Analysis

Single-trial data are extracted from continuous data (.eeg file) using an event file (.pos) for one subject. In the time domain, emergence of responses relative to a baseline can be performed using Wilcoxon paired-value tests (or parametric *t*-tests), and conditions can be compared using Kruskal-Wallis tests or randomization tests. Similar computations can be made in the time-frequency domain (with tests performed for each sample and each frequency for each channel), with the addition of randomization statistics to test inter-channel synchrony (example in Figure 6).

#### 2.4.2. Across-Subject Statistics for Group-Level Analysis

Event-related responses (.p file) in the time domain can be compared between conditions for a group of subjects using Wilcoxon paired-value tests (example in Figure 4), Quade tests, or randomization tests, and compared between groups of subjects using Kruskal-Wallis tests. In the time-frequency domain, paired conditions can be compared for a group of subjects using Wilcoxon or Quade tests.

### 2.5. Interoperability and MATLAB Compatibility

ELAN is fully compatible with MATLAB. Input and output functions (read/write) for data import and export from and into MATLAB are available for all three ELAN data formats (e.g., eeg2mat.m, mat2tf.m, mat2ep.m, etc.). In addition, ELAN can also export data to several MATLAB-based toolboxes such as SPM, FieldTrip, and Nutmeg. Data export functions for other toolboxes such as EEGLab and BrainStorm as well as a range of additional import routines will be available soon. 

## 3. Discussion

### 3.1. Advantages and Specificities of ELAN

ELAN is a toolbox which allows to analyze virtually any kind of electrophysiological continuous signal (EEG, MEG, iEEG, ECoG, LFP, etc.). ELAN results from 25 years of interactions between methodologists, software developers, and users, which has resulted in a robust and useful set of functions covering a wide range of applications in neurophysiology. Regarding the type of data processing and analysis that ELAN can perform, its main strengths are an easy preprocessing of the data, advanced scalp-level topographical mapping, time-frequency analysis, and adequate nonparametric and randomization statistics. Computations are run with command-line programs which are easily scriptable. Two of the main advantages of ELAN are the user-friendly navigation in the acquired and processed data which is made possible through three interactive viewers (*EEG*, *ERPA*, and *TFVIZ*) on one hand and the fast computations on the other hand. The high processing speed is achieved through optimized algorithms and compiled C code. This is particularly important for time-frequency analysis and related statistics. Several computation-intensive procedures can further be run using distributed computing according to the MPI standard. In addition to importation routines to read data from many commercial EEG or MEG systems, I/O MATLAB functions are available for all ELAN data formats, and we are currently adding I/O functions for other analysis toolboxes which offer complementary functionalities such as source modeling (SPM, BrainStorm, Fieldtrip, etc.). Future implementations of additional functions optimized for real-time analysis are currently under development. The speed of ELAN code written in C would be ideal for real-time applications that require online processing of electrophysiological signals, such as brain-computer interface (BCI) systems. Furthermore, by contrast to a number of alternative freely available toolboxes, ELAN does not require the purchase of a MATLAB license. In addition to being a significant argument for some laboratories with limited financial resources, the fact that ELAN is independent of MATLAB also means that it is not affected by changes occurring between different MATLAB releases. In fact, backward compatibility within ELAN is an important aspect of our development policy. Finally, as mentioned above, a further specificity of ELAN is the availability of a simple and straight-forward tool for manual sleep stage scoring based on polysomnographic data (i.e., combination of EEG, EMG and EOG signals acquired during sleep).

### 3.2. Current Limitations

In terms of data analysis, one current limitation of ELAN is an absence of source-reconstruction programs for EEG/MEG. However as mentioned above, it is possible to export data which have been pre-processed with ELAN to other software packages that have source-modelling procedures. Reciprocally, the resulting source time series obtained with other toolboxes can be imported into ELAN for further analysis (a template MATLAB function for conversion from  .mat to  .eeg file format is provided with ELAN). For example, source-level time series estimated using BrainStorm or SPM can easily be imported into ELAN for time-frequency analysis and statistical testing. Note also that ELAN libraries and header files are made available within the ELAN distribution package, with limited documentation. Moreover, upon user request, specific conversion files for specific source-level data formats (obtained with other toolboxes) could be made available. ELAN is currently not open-source because the readability of the source code needs to be improved (i.e., cleaning up variable names, improving interpretability and architecture of the code). Nevertheless, it is already easy to achieve a reasonably flexible level of customization of the toolbox based on MATLAB interfacing or independent C routines, reading and writing ELAN file formats.

### 3.3. Software Access and Support

The ELAN software package is available for download free of charge through the ELAN web platform at http://elan.lyon.inserm.fr/. A detailed documentation is available for all ELAN routines, either on the website or as a pdf file. Although only limited online support is currently provided, we aim to enhance support especially through the creation of a community of users that exchange information through a dedicated forum on the ELAN website. We expect that the combination of continued in-house development and a growing knowledge base will enhance the quality, flexibility, and user-friendliness of ELAN. A user mailing list and ELAN newsletter also provides ELAN users with up-to-date information on software updates, availability of additional plug-ins, and bug reports. One of the future plans is to release ELAN source code in order to promote new developments and exchange of expertise in the community. Most importantly, we will continue to enhance the interoperability of ELAN with other free toolboxes in order to strengthen interactions and foster scientific cross-fertilization. We hope that ELAN will be an active contributor to the growing common effort within the community of method developers, programmers, and neuroscientists to substantially improve the power and reliability of neurophysiological data analysis tools over many years to come.
